# Supplementary material for: Conditional chemoconnectomics (cCCTomics) as a strategy for efficient and conditional targeting of chemical transmission
Source: eLife. 2024 Apr 30;12:RP91927. doi: 10.7554/eLife.91927 (PMC11060718; doi:10.7554/eLife.91927)
Supplement: Figure 3—source data 3. [file elife-91927-fig3-data3.zip › Figure 3 source data3 part 1.zip!/Figure 3-Source data 3-part1/1063-13-CRISPResso_Report_8cz_fsdr.zip!/CRISPResso_on_1063_15/3b.Insertion_deletion_substitutions_size_hist.pdf]

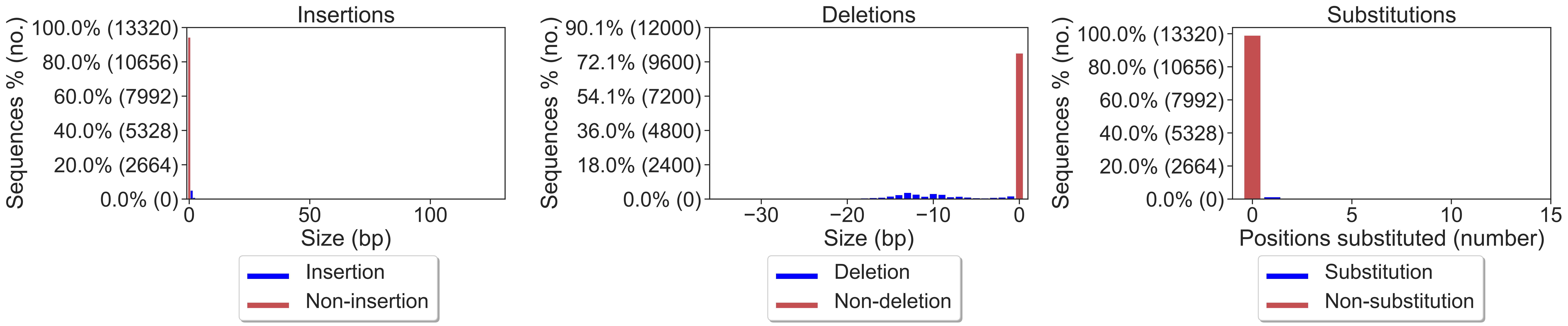

Insertions
Sequences % (no.)
100.0% (13320)
80.0% (10656)
60.0% (7992)
40.0% (5328)
20.0% (2664)
0.0% (0)
0
50
100
Size (bp)
Insertion
Non-insertion
Deletions
Sequences % (no.)
90.1% (12000)
72.1% (9600)
54.1% (7200)
36.0% (4800)
18.0% (2400)
0.0% (0)
−30
−20
−10
0
Size (bp)
Deletion
Non-deletion
Substitutions
Sequences % (no.)
100.0% (13320)
80.0% (10656)
60.0% (7992)
40.0% (5328)
20.0% (2664)
0.0% (0)
0
5
10
15
Positions substituted (number)
Substitution
Non-substitution
